# Editorial Changes

**Published:** 1855-03

**Authors:** 


### EDITORIAL CHANGES.

The editorial duties of The Boston Medical and Surgical Journal, have been assumed by Drs. WM. W. MORLAND and FRANCIS MINOT, who succeed Dr. GEORGE STEVENS JONES, resigned. These gentlemen are favorably known to the profession and will labor to increase the attractiveness of the Journal, and forward the interests of our common calling.

---

Drs. D. J. CAIN and F. PEYERS PORCHER, have retired from the editorship of the *Charleston Medical Journal and Review*, and their places are filled by Dr. C. HAPPOLDT. We regret to part company with such dignified and pleasant confréres as Drs. C. and P., and their loss will be widely felt by the profession.

---

Dr. BENNET DOWLER, whose connection with The New Orleans *Medical and Surgical* has just expired, purposes issuing a Quarterly Journal, to be entitled "The New Orleans Quarterly Journal of Medicine.

